# Establishment of a culture model for the prolonged maintenance of chicken feather follicles structure *in vitro*

**DOI:** 10.1371/journal.pone.0271448

**Published:** 2022-10-07

**Authors:** Corentin Mallet, Laurent Souci, Mireille Ledevin, Sonia Georgeault, Thibaut Larcher, Caroline Denesvre

**Affiliations:** 1 ISP, INRAE, Université de Tours, Nouzilly, France; 2 INRAE, Oniris, PAnTher, APEX, Nantes, France; 3 Plateforme IBiSA des Microscopies, Université de Tours et CHRU de Tours, Tours, France; University of Colorado Boulder, UNITED STATES

## Abstract

Protocols allowing the *in vitro* culture of human hair follicles in a serum free-medium up to 9 days were developed 30 years ago. By using similar protocols, we achieved the prolonged maintenance *in vitro* of juvenile feather follicles (FF) microdissected from young chickens. Histology showed a preservation of the FF up to 7 days as well as feather morphology compatible with growth and/or differentiation. The integrity of the FF wall epithelium was confirmed by transmission electron microscopy at Day 5 and 7 of culture. A slight elongation of the feathers was detected up to 5 days for 75% of the examined feathers. By immunochemistry, we demonstrated the maintenance of expression and localization of two structural proteins: scaffoldin and fibronectin. Gene expression (assessed by qRT-PCR) of NCAM, LCAM, Wnt6, Notch1, and BMP4 was not altered. In contrast, Shh and HBS1 expression collapsed, DKK3 increased, and KRT14 transiently increased upon cultivation. This indicates that cultivation modifies the mRNA expression of a few genes, possibly due to reduced growth or cell differentiation in the feather, notably in the barb ridges. In conclusion, we have developed the first method that allows the culture and maintenance of chicken FF *in vitro* that preserves the structure and biology of the FF close to its *in vivo* state, despite transcriptional modifications of a few genes involved in feather development. This new culture model may serve to study feather interactions with pathogens or toxics and constitutes a way to reduce animal experimentation.

## Introduction

Feathers are the most complex hard skin appendage to have been produced during vertebrate evolution [1, 2]. At the beginning, in Pterosaurs and Dinosaurs, filamentous feathers covered most of the body [3] and probably ensured essential functions such as thermoregulation, body protection or mate attraction. Later, during avian evolution, the development of vaned feathers was linked to flight. Structurally, juvenile and adult contour feathers are made of a rachis (*i*.*e*. shaft) branched with barbs and barbules. Each feather is anchored through its extremity, the calamus, in a small cavity invaginated in the thickness of the skin, called the feather follicle (FF). The FF, the mini-organ producing the feather, cycles between two phases: the growing phase, when the feather is generated, and the resting phase, when the feather has reached its appropriate size [4–6]. Indeed, unlike the human hairs that could grow over a long period of time (anagen phase, up to 7–8 years), feathers grow only during embryogenesis or upon regeneration, after natural molt (i.e., growth of juvenile feathers replacing downy feathers), or after plucking [6, 7].

**Fig 1:**
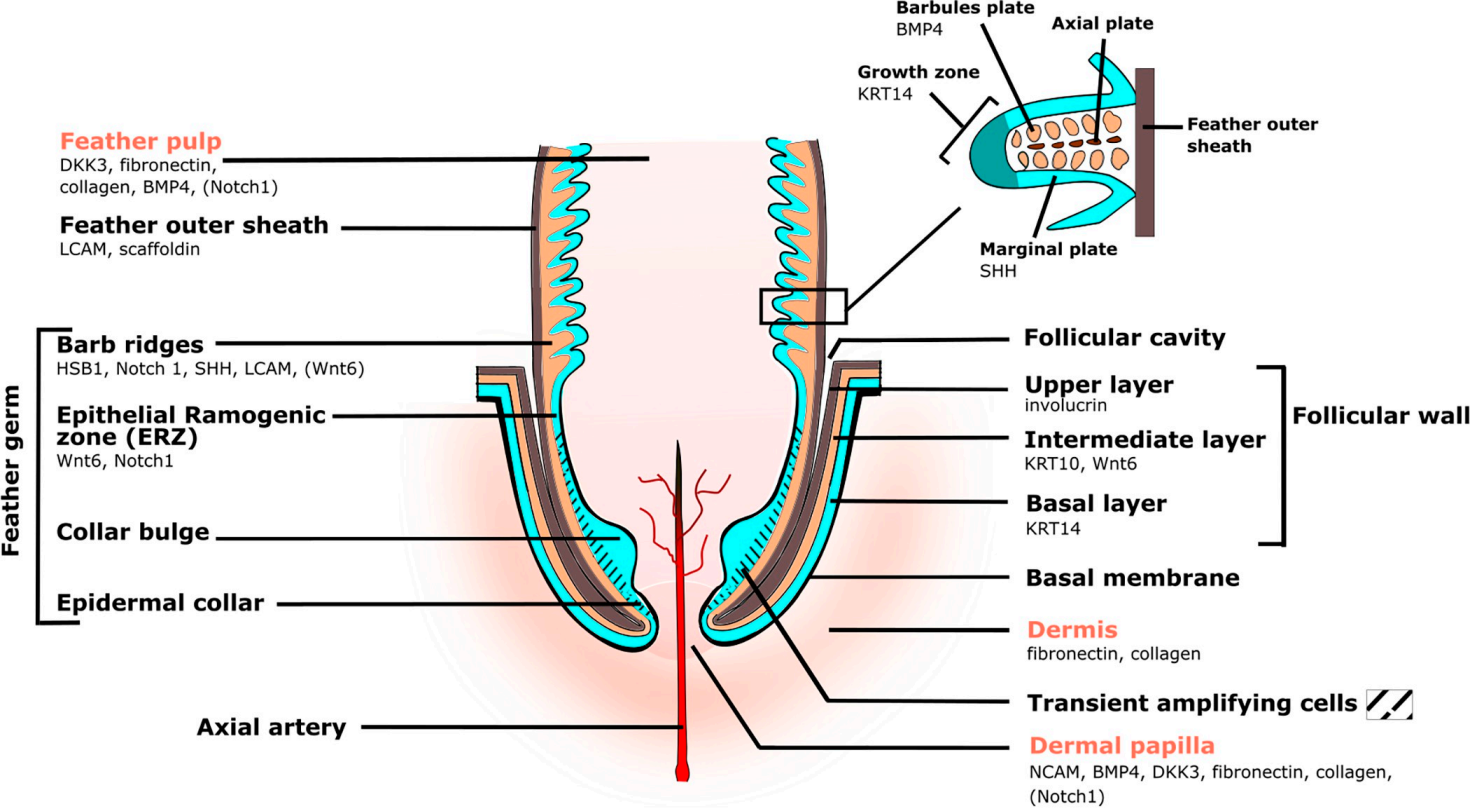
Schematic representation of a feather follicle with a growing feather. Adapted from [4]. Legends for the mesenchymal compartment appear in pink. It includes the dermis, the dermal papilla, and the feather pulp. Legends for the epidermal compartment appear in black. The epithelia (follicular wall and feather epithelium) are made of three layers: the basal layer (in blue), the intermediate layer (in orange), and the upper layer (in brown). The feather outer sheath surrounds and protects the feather germ. The feather germ epithelium is composed of four structures from the bottom to the top: i) the epidermal collar, ii) the collar bulge, iii) the epithelial ramogenic zone (ERZ), and iv) the barb ridges. Transiently amplifying cells (hatched area) are localized in the proliferative zone. The basal layer involuted into barb ridges is called the marginal plate. As shown in the enlargement (black frame) of one barb ridge, the basal layer/marginal plate proliferates at barb ridges extremity, thus providing numerous barbs and/or barbules cells. This allows the formation of the axial and barbule plates which give rise to barbs and barbules, respectively. The space between the follicular wall and the feather outer sheath is the follicular cavity. The feather follicle is irrigated by a prominent axial artery. Gene markers expressed in various zones of the FF and used in this study are indicated below each corresponding structure.

The FF structure and the feather formation have been studied for many years, in regards to their development, comparative biology, and evolution/paleontology (reviewed in [3–5, 7, 8]). The FF is constituted by an epidermal component and a mesenchymal component (which have distinct embryologic origins), separated by a basement membrane [9]. The mesenchymal component includes the dermal papilla and the feather pulp (Fig 1). The dermal papilla at the basis of the FF acts as the control center of feather growth and regeneration. The feather pulp is organized as a loose extracellular matrix and is only detectable in growing and differentiating feathers [6, 10]. The feather pulp is a highly vascularized zone irrigated by the axial artery that provides nutrients to the growing feather through the blood. The epidermal part of the FF corresponds to an epidermis folded on itself and invaginates into the dermis of the skin. At its basis, the FF epidermis comprises a proliferation zone, with the epidermal collar above the dermal papilla and the collar bulge, which hosts the FF stem cells (Fig 1) [11]. This epithelial zone regenerates the feather under the influence of the dermal papilla [11]. Upper in the FF are the epithelial ramogenic zone, where the formation of barb ridges is initiated into the epidermal cylinder, and the barb ridges themselves (Fig 1) [4–6, 11]. The feather epithelium contains three layers (Fig 1): (i) The innermost layer corresponds to the basal layer, and is constituted by undifferentiated keratinocytes with high dividing potential. In more distal regions, the keratinocytes proliferative ability is progressively lost and the basal layer becomes cornified forming the feather inner sheath. (ii) The intermediate layer is constituted of keratinocytes that have differentiated between the proliferation zone and the barbs ridges level, and from which will originate most parts that constitute a feather (barbs, barbules, rachis, and calamus) [7]. (iii) The outermost layer is a cornified layer, surrounding the basis of the growing feather, named the feather outer sheath (Fig 1). This outer sheath disintegrates when the feather pops up.

Feathers and hairs show obvious differences in macroscopic morphology, but also in protein composition (*e*.*g*., corneous beta-proteins (CBP) are cystein-rich keratins specific of birds and are absent in mammal hairs) and anatomy (*e*.*g*., sebaceous glands are absent in FF) [2, 12–14]. Besides anatomical and structural differences, FF and hair follicles (HF) express similar genes at the early stages, including genes involved in morphogenesis (Shh, Wnts, and BMPs), albeit at different times and locations [4, 15–18]. They also exhibit homologies in physiology, function, and as self-renewal mini-organs involving stem cells [16, 19]. Protocols for the maintenance of human HF have been previously described. Westgate et al. first reported the successful maintenance of microdissected human HF cultivated *in vitro* for at least 9 days in a defined serum-free medium [20]. In these conditions, 90% of the hairs continued to grow [20]. Since then, HF culture has allowed tremendous progress in hair biology and dermatology, as well as in the fields of pharmacology or cosmetology (for review, see [21, 22]).

No protocol exists to date for the maintenance of FF in culture. Such a protocol could bring new insights in feather development and in comparative biology between skin appendages. Moreover, such a protocol will also be an interesting *in vitro* model to study the interactions between feathers and micro-organisms/pathogens. In that perspective, and because of the homologies shared between chicken FF and human HF highlighted above, we hypothesized that previous cultivation conditions depicted for HF might be suitable for FF. Herein, we explored the *in vitro* maintenance of FF microdissected from juvenile chickens by adapting the Westgate’s protocol. This protocol was chosen because it was very simple to implement and not finely adapted for human hair cultivation yet. With this protocol, we succeeded in maintaining chicken FF in culture for 7 days and even grew them. This provides a novel *in vitro* integument culture model for the chicken.

## Materials and methods

The SPF White Leghorn chickens used in this study were provided by the infectiology platform (PFIE) of INRAe (Tours-France) (https://www6.val-de-loire.inrae.fr/pfie_eng/). The chickens were offsprings from breeder replacement hatchs of SPF WL population. The chickens were bred until and euthanized at the PFIE platform, according to protocols and procedures approved by the Departemental Directorate for the Protection of Populations (DDPP), for the French Ministry of Agriculture and Food (Agreement #D-37-175-3). The PFIE is part of the international network VetBioNet (2017-).

### Animals

**Fig 2:**
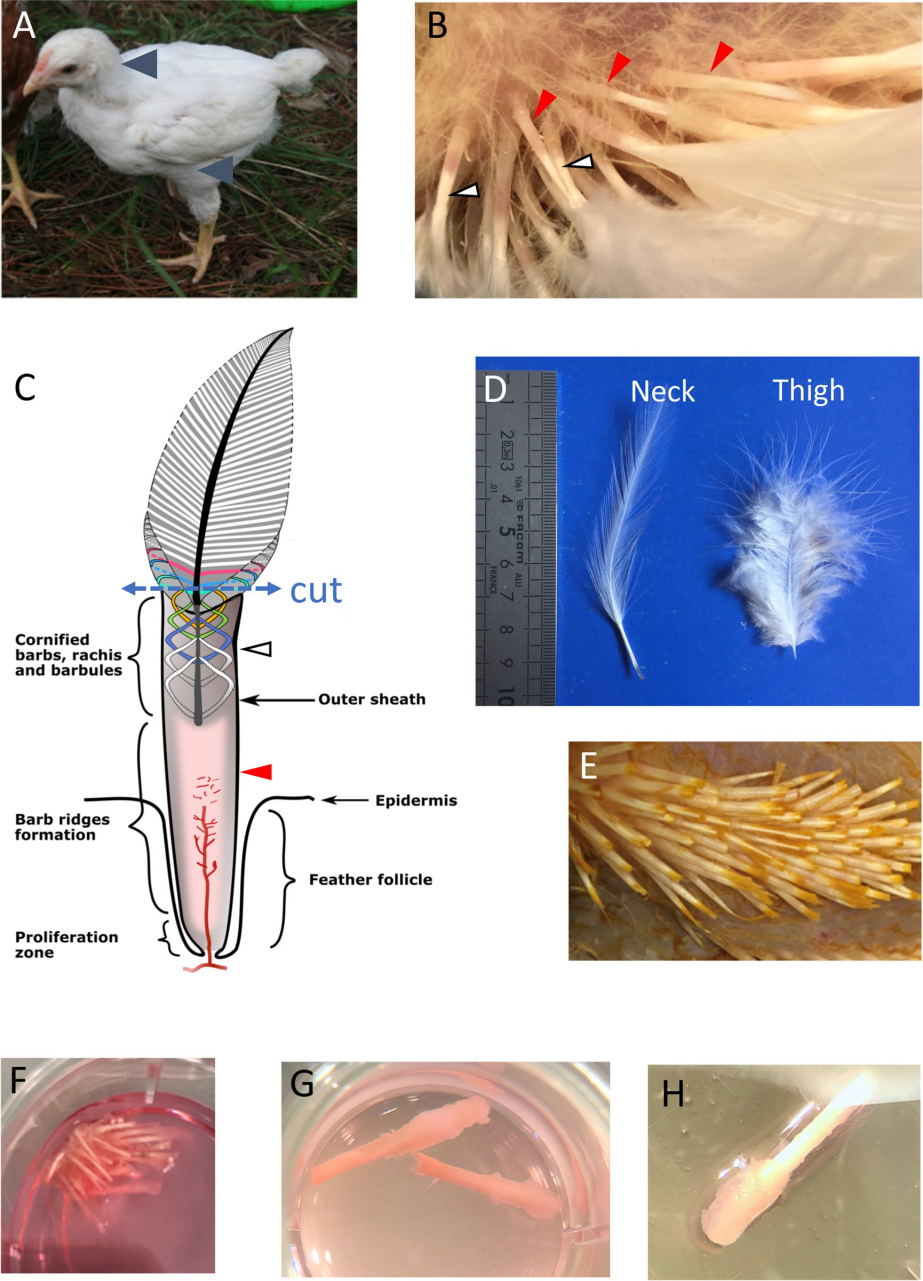
Steps to obtain individual feather follicles for *in vitro* culture. In 3-week old White Leghorn chick (A), the contour feathers from two regions (blue arrowheads) (the base of the neck or the thigh) have reached a developmental stage (B, C) before becoming adult contour feathers (D). Note that feathers are heterogenous in their development (B), with feathers just popping-out the outer sheath (red arrowhead) or feathers close to their terminal size. The white zone (white arrowheads) corresponds to cornified rachis containing barbs and barbules (devoid of pulp), which are limited by the outer sheath (B, C); in contrast the pink zone (red arrow) corresponds to live tissue (B). Skin pieces (E) after cutting at the growing feathers (as shown in C, with blue dotted arrow) were soaked into supplemented William’s E medium (see text) (F), and then progressively dissected into individual growing follicles (G, H).

Specific pathogen-free White Leghorn chicks (B13/B13) were obtained from INRAE Val-de Loire animal facilities, that has an agreement to rear and euthanize birds (agreement C37-175-4 of 20 december 2016, delivered by the “prefecture d’ Indre-et-Loire”, France). Four males between 22 and 26 day-of age (weight of 180–230 g) were used for the entire study and were offspring from the biannual breeder-replacement flock. They had their neoptile feathers (down) replaced by the first generation of teleoptile feathers (juvenile feathers). Animals were bred in group in a controlled and enriched environment (temperature, with a 12:12 L:D lighting scheme with food and water available *ad libitum*) until euthanasia by cervical dislocation according to the guidance and regulation of the French Ministry of Higher Education and Innovation (MESRI) with appropriate staff and good practices. After cutting the feathers 0.5cm above the skin at the outer sheath level (Fig 2C), the feathered skin from the back of the neck and the external upper part of the thigh were properly disinfected using 10% betadine solution. Feathers were cut to avoid microorganism contaminations and allow cultivation in closed microwells. Skin pieces of about 5cmx2cm were cut and immerged into William’s E medium (#32551–020, Gibco) supplemented with fungizone (2.5μg/mL) (#A2942, Sigma-Aldrich) and penicillin/streptomycin (100 UI/mL each) (DE17-602E, Lonza). These two body zones were chosen given their density in first juvenile feathers of average size and a low fat subcutaneous tissue. Importantly, feathers showed heterogeneity (Fig 2B), some just popping-up out the outer sheath and others more advanced in their growth and differentiation, even cornification. Even if we did our best to select FF with growing feathers based on morphological aspect, this was not warrantied for all FF, because of the cut of feathers extremity at skin samplings.

### Antibodies

The antibody to fibronectin (mouse IgG2a, B3/D6, developed by M. Fambrough) was obtained from the Developmental Studies Hybridoma Bank, DSHB, maintained by The University of Iowa, USA. A rabbit polyclonal antibody recognizing several alpha-keratins (Pan-cytokeratin) was used (Signet labs #468–26). The mouse antibody to scaffoldin was generously provided by Mlitz et al. [23].

### Isolation and maintenance of feather follicles in culture

After cutting the skin pieces into smaller ones, the isolation of FF was performed as previously described for HFs [24]. Briefly, each FF was microdissected individually by using scalpel blade and forceps under a stereomicroscope (MZ8, Leica). The maximum amount of fat was removed around the FF bulb, with great care of keeping the FF bulb morphologically intact. Isolated FF were deposited into William’s E medium supplemented with penicillin/streptomycin, 10 ng/mL of hydrocortisone (#H0396, Sigma-Aldrich) and 10 μg/mL of recombinant human insulin (#0219390080, MP Biomedicals). Isolated FF were maintained free-floating in individual well of 24-well multi-plates, with two to three isolated FF depending of their diameter, in 500 μL culture medium (described above) per well. Plates were incubated at 37°C as reported earlier for chicken embryo skin explants [25–27], in an atmosphere with 6% CO2. The culture medium was changed every 2 days.

### Morphology and feather growth

The morphology of the FF was examined macroscopically every day. Pictures were taken on fresh FF through one ocular lens (a eye piece) of the stereomicroscope with an iphone8 (Apple, France). Feather growth was determined on fresh FF placed on a graph paper and pictured every 2 to 3 days for 11 days. The size was next determined graphically after pictures enlargement from the base of the FF to the top of the cut feather. After each measure, FF were put back in culture until the next measure or the end of the experiment.

### Histology and immunochemistry

Two FF were taken out from culture at each time point of the kinetic (in 2 independent experiments) and were fixed with 4% formalin, embedded in paraffin wax, longitudinally cut into 4 μm serial sections [28]. A first section was stained using a routine hematoxylin-eosin-safranin (HES) staining method. At least two sections of each sample were observed by a skilled pathologist; tissue and cell alterations were systematically recorded. A second section was used to visualize collagen with a trichrome Masson staining. Additional sections were used for immunochemistry using the following primary antibodies: anti-pancytokeratin (1:40), anti-fibronectin (1:1000) and anti-scaffoldin (1:2000). Briefly, deparaffinized sections were incubated in 10 mM citrate buffer (pH 6, Dako) for 40 min at 98°C. The sections were then incubated with the Peroxidase solution (3%) for 10 min and saturated with BSA (2%) for 30 min at room temperature. After incubating tissue specimens with the primary antibody overnight at 4°C, and then with a specific biotinylated secondary antibody (1:300, Dako) for 30 min at room temperature, immunoreaction complexes were revealed using streptavidin-coupled peroxidase (1:300, Dako) and DAB chromogen (Dako). Sections were then counterstained with Gills’s hematoxylin and observed with a Nikon microscope (Eclipse Ni) and a Nikon DS-Ri color-camera.

### Transmission electron microscopy (TEM)

FF were fixed in 1% glutaraldehyde, 4% paraformaldehyde, (Sigma, St-Louis, MO) in 0.1 M phosphate buffer (pH 7.2). Samples were then washed in phosphate bufferand post-fixed by incubation for 1 h with 2% osmium tetroxide (Agar Scientific, Stansted, UK). Samples were then fully dehydrated in a graded series of ethanol solutions and propylene oxide. They were progressively impregnated with 3 mixtures of propylene oxide/Epon resin (Sigma) and finally left overnight in pure resin. Samples were then embedded in Epon resin (Sigma), which was allowed to polymerize from 37°C to 60°C. FF were initially sectioned at 600 nm of thickness, in the longitudinal plan from the external part to the center, and stained with toluidine blue to locate the level of cut. At each level, ultra-thin sections (50 to 80 nm) were next performed with a Leica EM UC7 ultramicrotome (Wetzlar, Germany). Ultrathin sections were stained with 5% uranyl acetate (Agar Scientific), 5% lead citrate (Sigma), and observations were made with a transmission electron microscope (JEOL 1011, Tokyo, Japan) as previously [29].

### RNA isolation from feather follicles and RNA expression quantification by reverse transcription qPCR (RT-qPCR)

FF were taken out from culture at each time point of the kinetic snapped frozen and stocked at -80°C before RNA extraction. Each sample made of two FF were lysed into RLT lysis buffer (Qiagen) with extemporaneous addition of 1% ß-mercaptoethanol in presence of ceramic beads (#19-645-3 OMINFIT) by mechanical agitation until biological material disappearance. RNAs were next extracted from the supernatant by using the RNeasy minikit (#74104, Qiagen), according to the manufacturer’s recommendations. RNAs were treated with Rnase-free RQ1 Dnase (#M6101, Promega) and RNAs concentrations were measured with a Nanodrop spectrophotometer. Four hundred fifty ng of total RNA was reverse-transcribed into cDNA by using Moloney MLV reverse-transcriptase (#M1701, Promega) with 50μg/ml oligo(dT) primers (#C110A, Promega) and dNTP (#U151A, Promega). The expression of genes of interest was next measured by real-time quantitative PCR (qPCR) in triplicate with iQ Supermix SYBR green (#1708882, Bio-Rad) on a C1000 Touch CFX96 Real-Time System (Bio-Rad). The primer used for the qPCRs were synthetized by Eurogentec (Table 1). The qPCR program consisted of a 5 min activation step at 95°C, followed by 40 cycles of 95°C for 20 sec and 60°C for 35 sec. For each marker, qPCR results were obtained from three different samples (with 2 FF/sample) of two independent experiments. The chicken ribosomal protein S17 gene (RPS17) was used as the reference housekeeping gene as reported previously [30]. The mRNA levels of each gene after dissection (Day 0) was normalized to the expression of the RPS17 by the 2^-ΔCt^ and expressed in an arbitrary unit (A.U), the RPS17 expression being set at 1 A.U. Next, to define the fold-changes in mRNA expression for each gene of interest between Day 0 and the different time points of cultivation, the calculation was done as follow: first, for each experiment, the mRNA gene expression at Day 0 was calculated relatively to the expression of RPS17, by using the Ct mean of each replicate (1 value per sample). The relative expression of each interest gene (in fold change) after 3, 5 or 7 days of culture was determined by the 2^-ΔΔCt^ threshold cycle (Ct) method for each replicate (3 samples in triplicate), with the expression at Day 0 being set at 1, as reported previously [30].

**Table 1:** Primers used for qPCR in this study.

Gene name	Forward Primer (5’-3’)	Reverse Primer (5’-3’)	References
Notch1	GGCTATTCCTGTGAGTGCGT	CTTGAGTTCCTCTGGGGCAG	NM_001030295
BMP4	GCTGATATGCCTTGCTTGCT	ACTTTCTTCCTGCCGGTCTC	MH553646
Shh	CTGTCTCCCGACCAAACTCC	CCACCGATCCCTAGCAAGAC	NM_204821
DKK3	TGAAGTCTGAGCATGACCCG	GCACGAAAACGGATGCTCAA	NM_205125
Wnt6	GCGACAACATCAAACCTCCC	TTGGCAGAGCAGAAATCGGG	NM_001007594
KRT14	GCGAGGACGCCCACATCTCTTC	TGAGCGCCATCTGCTCACGG	NM_001001311
LCAM	GTGGAGAACAAAGTGCCCCT	GATAGGGGGCACGAAGACAG	NM_205153
NCAM	ACGGAACGGCTATTTCGGAG	ACGCTGATCTCTCCCTGACT	NM_001128828
HBS1	TGGCCCTGGACATTGAGATT	TGGCTCCAGTCTTCACAGAG	[14]

### Statistical analysis

All graphs and statistical analysis were performed using GraphPad Prism software version 8 (San Diego, USA). Data are presented as median and interquartile range. Kruskal-Wallis test with a Dunn correction for multiple comparison was used. Adjusted p-value <0.05 were considered statistically significant as indicated in figure legends.

## Results

### The feather follicle epithelium is extensively well preserved when cultivated *in vitro* for 7 days

**Fig 3:**
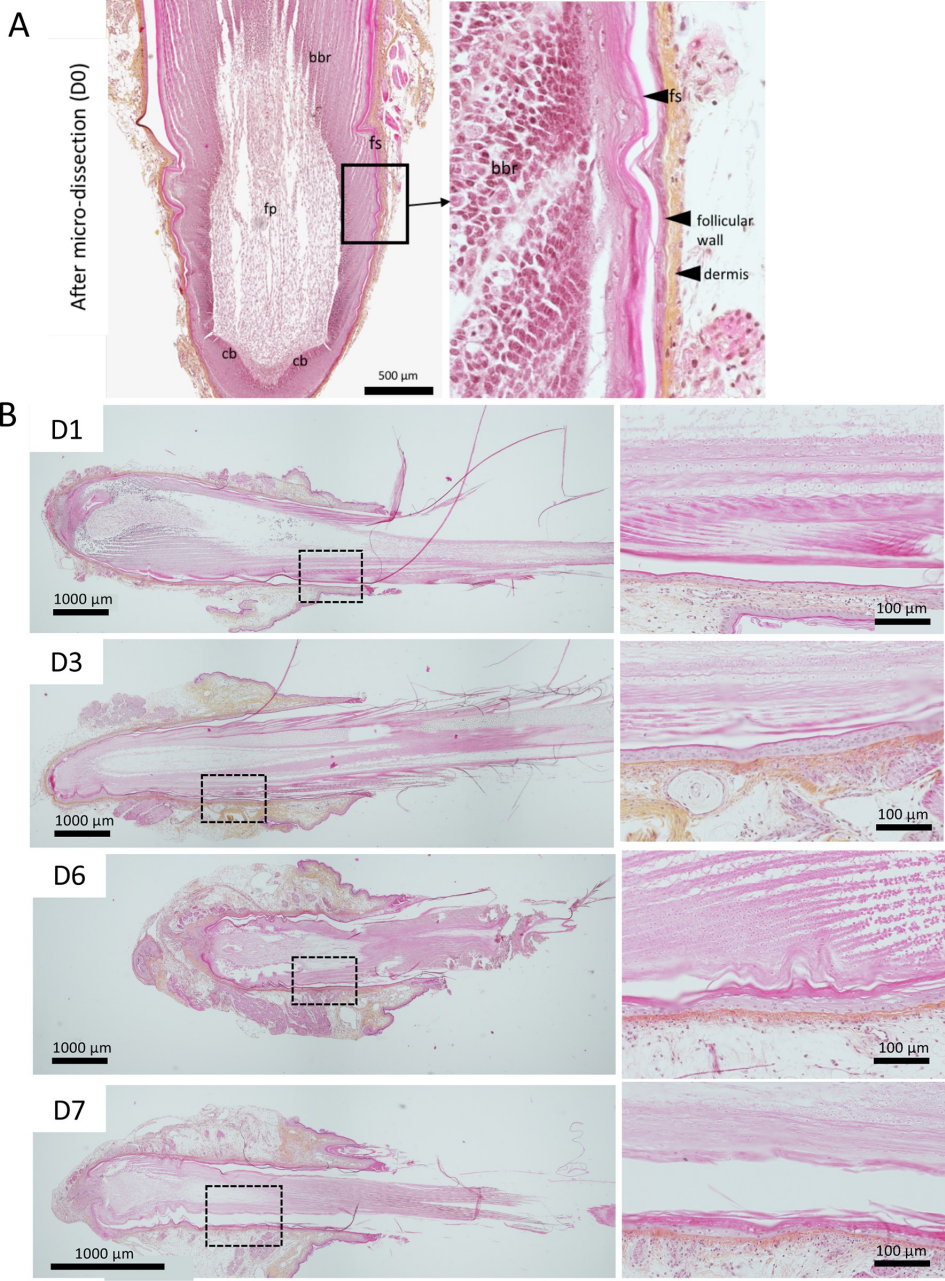
Histological structure of feather follicles from microdissection to Day 7 of *in vitro* cultivation. Formalin-fixed FFs embedded in paraffin were sectioned and stained with HES. (A) Overview of a typical FF with a growing feather after microdissection. bbr, barb ridges; cb, collar bulge; dermis; fs, feather sheath; follicular wall; fp, feather pulp. An enlargement of the barb ridges is shown. (B) Structure of FF after 1 (D1), 3 (D3), 6 (D6), and 7 (D7) days of cultivation *in vitro*. A low magnification showing the whole FF with its feather (left panels). An enlargement of the barb ridges (right panels).

**Fig 4:**
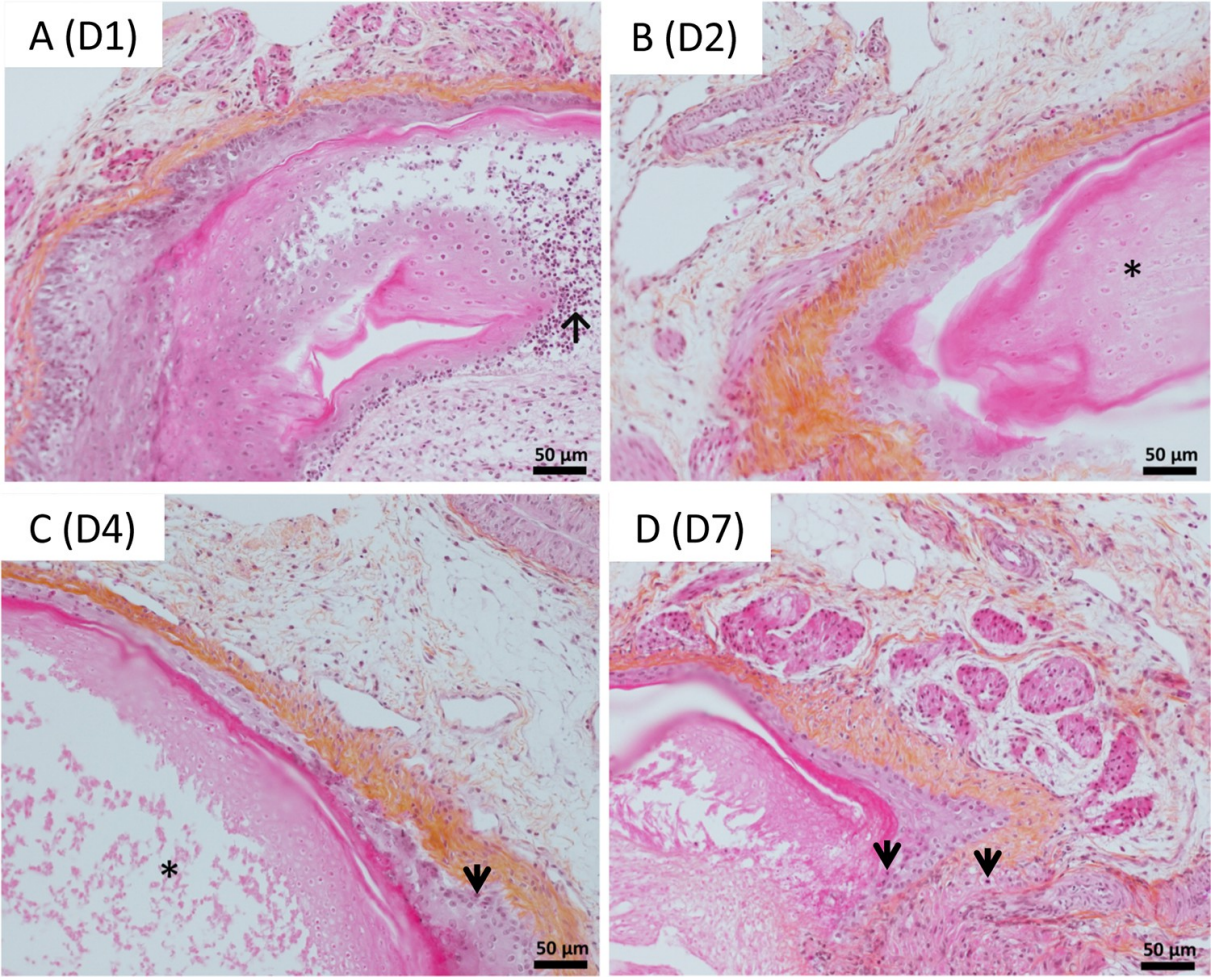
Example of lesions observed in the cultivated FF from 1 to 7 days. The black arrows indicate pyknotic nuclei. The asterisks (*) indicate necrotic feather pulp without viable cells visible (incl. “ghost” cells at Day 2).

Chicken juvenile feathers replacing downy feathers were microdissected from two body zones, the basis of the neck and the external face of the upper thigh. Most of the truncated feathers associated to the FF dissected in that study harbored growing feathers at different stages of growth (Fig 2B). A schema of the experimental protocol is shown in Fig 2. FFs with their truncated feather were maintained in culture in groups of 2 or 3, free-floating in the medium. To assess the tissue preservation and architecture of the feather and follicular wall, FFs cultivated for 1 to 7 days were collected every day (except at day 5) and examined microscopically after longitudinal sectioning and staining with HES. At low magnification, FFs showed a typical morphology of growing feather as previously described [31] after microdissection (Fig 3A) and after cultivation (Fig 3B, left panel). Based on the barb ridges morphology in the FF, most of the feathers examined were still growing (Fig 3B, right panel) at 1, 6 or 7 days in culture. In contrast, the FF cultivated for 3 days showed a feather with cornified rachis, barbs, and barbules (Fig 3B, D3). This feather had probably already stopped its growth when microdissected. Next, a careful examination of FFs and feathers was performed to look for evidence of necrosis (Fig 4). After 1 day of culture, cell dissociation and hyperchromatic condensed (pyknotic) nuclei were visible in the barb ridges, near the dermal papilla and at the basis of the feather pulp (Fig 4A). At Day 2, nuclear debris had essentially disappeared from the barb ridge and the feather pulp, leaving only “ghost” cells, with preserved borders but a pale hyaline cytoplasm and a fainted barely stainable nucleus (Fig 4B). From Day 3 to Day 6, the follicular wall was preserved and was rarely damaged; the type of damage observed was, for instance, cells with an abundant and a clear cytoplasm corresponding to balloonisation or dead cells with a pyknotic nucleus (Fig 4C). At Day 7, about 10–15% of the cells of the FF epithelium (follicular wall) showed evidence of necrosis (pyknotic nucleus and/or ghost cells) (Fig 4D).

A second experiment was performed similarly with a prolonged cultivation of the FFs over 13 days in order to determine the longest time of culture preserving the organ’s structure. The microscopic observations performed at 3, 5, and 7 day of culture gave results similar to that above (S1 Fig). At the latest time points (Day 11 and 13), a progressive increase in the number of pyknotic nuclei in the follicular epithelium, dermal papilla, and dermis connective tissue was observed. At Day 13, most of the epithelial cells were necrotic, a few post-necrotic mineralization deposits were visible, and only surrounding connective tissue of the dermis still appeared viable (S1 Fig).

Taken all together, these results indicated that the FFs displayed an essentially preserved architecture until Day 11. Although the feather pulp cells degenerated rapidly, the FF wall and the feather epidermis showed limited alterations until Day 7. Subsequent characterizations were therefore performed over 7 days of cultivation only.

### Limited ultrastructural changes were observed in the epithelia of the follicular wall up to 7 days of cultivation *in vitro*

**Fig 5:**
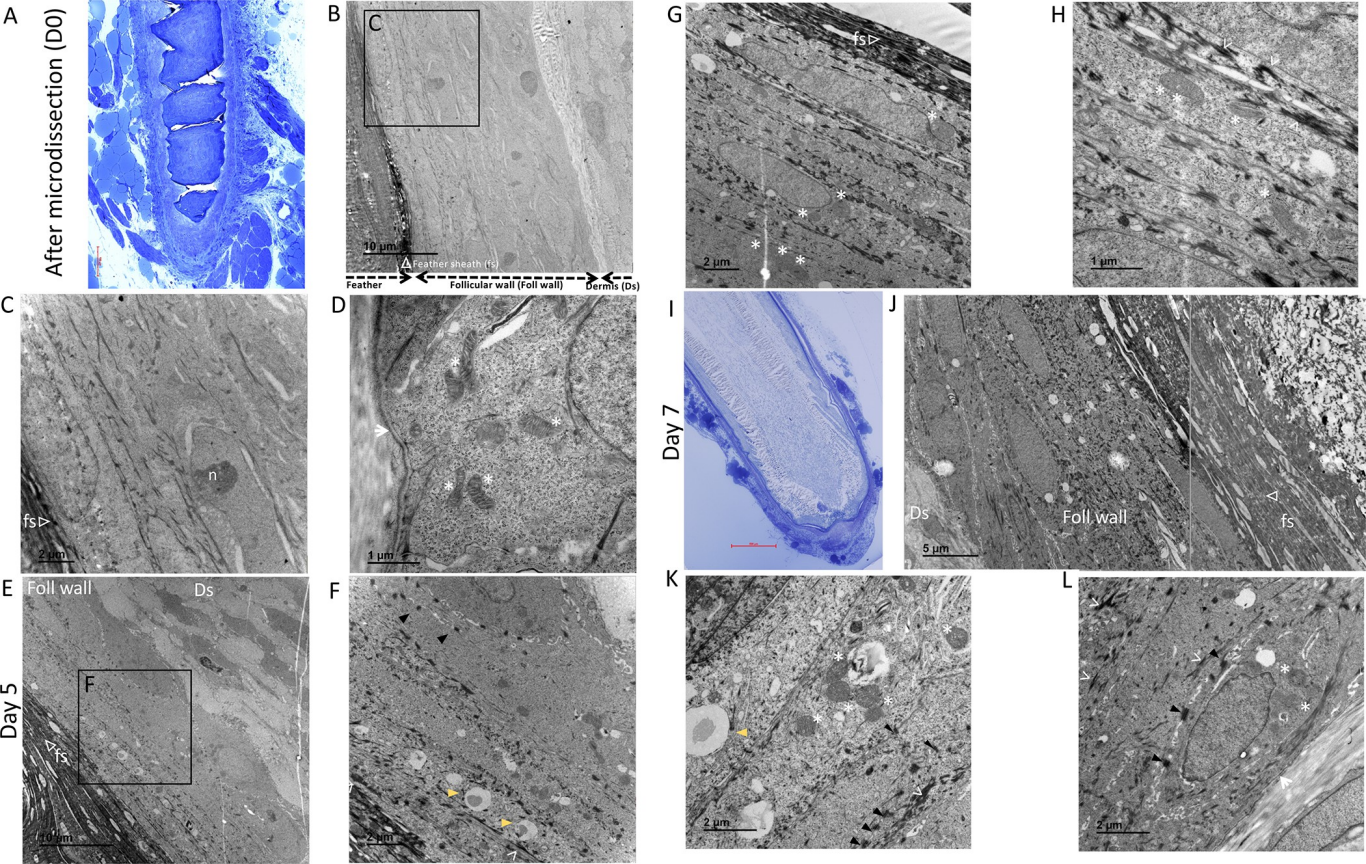
Morphology of the FF by TEM. Images of semi-thin section of a FF after microdissection (Day 0) (A) and at Day 7 (I), stained with toluidine blue observed by optical microscopy are shown and correspond to the level of the ultra-thin sections observed at these time points. Images of FF observed by TEM at different magnifications at Day 0 (B-D), Day 5 (E-H), Day 7 (J-L). Note that J was generated by merging two pictures. Regions enlarged in another picture are indicated with black frames. On panel (B), the dermis (Ds) and the follicular wall (foll wall) are indicated as well as part of the feather. A few structures are shown on the images: feather sheath (fs; empty white triangle), basal membrane (white short arrow), mitochondria (white asterisk symbol), nucleolus (n), keratin bundles (white plain arrowhead), desmosome (black plain triangle), lipid droplet (yellow plain triangle).

In order to refine the structure analysis of the FF stratified epithelium, we also examined the ultrastructure of the feather follicle by transmission electron microscopy (TEM). The observations were performed after microdissection (Day 0), at 5 and 7 days of culture, with a focus on the epithelium of the follicular wall and feather sheath (Fig 5). After microdissection, more than 6 layers of cells were observed in the follicular wall (Fig 5B), with two major types of epithelial cells: (i) in basal layers, large cells with a large nucleolus in the nucleus; (ii) in the upper layers, flat epithelial cells, rich in keratin bundles (Fig 5C). At higher magnification, the basal membrane was intact, and intracellular organelles (*e*.*g*., mitochondria and intercellular desmosomes) within epithelial cells were unchanged (Fig 5D). At Day 5, the epithelium was well preserved, with normal desmosomes observed (Fig 5F–5H). The major differences with freshly dissected FFs were the presence of lipid droplets or vacuoles in the upper layers and the absence of nucleoli in cells of the basal layers (Fig 5E and 5F). Numerous mitochondria were visible with a normal morphology (*e*.*g*. cristae) (Fig 5G and 5H). At Day 7, the epithelium remained intact with numerous desmosomes (Fig 5J–5L). However, some signs of cell damage appeared, such as small vacuoles in cells from all layers and swollen mitochondria with faded cristae lamellae (Fig 5K and 5L). No necrotic cells were found. Therefore, despite the presence of some signs of cell damage in the epithelium of the follicular wall at Day 7 of culture, the integrity of the epithelium was confirmed by TEM up to Day 7, in accordance with histological results.

### Feather growth and cell proliferation

**Fig 6:**
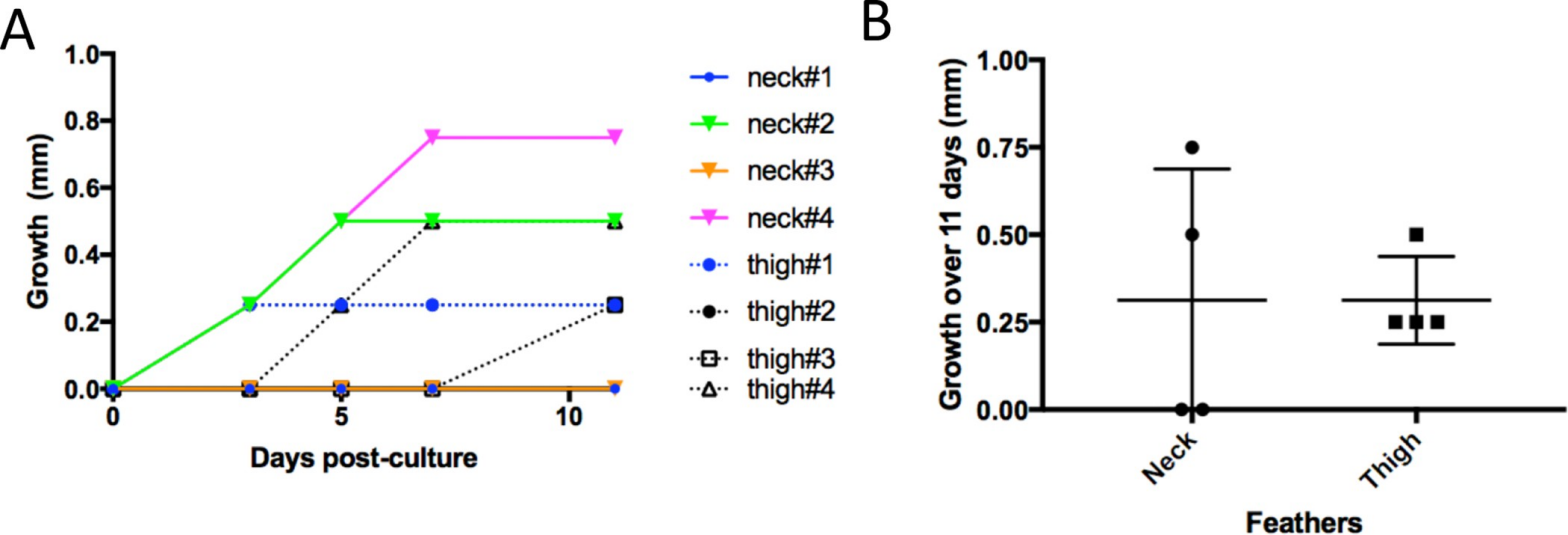
Growth of feathers cultivated *in vitro* for 11 days. The size (in mm) of 8 truncated feathers (4 from the neck and 4 from the thigh) inserted in their FF was measured after 3, 5, 7, and 11 days in culture. Note that each feather was shortened above the skin level before the dissection process. (A) Growth kinetic of individual feathers. Only the gain of growth over time is shown. Except for two feathers from the neck, all feathers weakly grew in length. (B). Total growth by origin (neck or thigh) over 11 days of cultivation.

To examine if growing feathers maintained in culture were still growing, we measured feather length on eight fresh juvenile feathers isolated from the neck (4) or the thigh (4) after dissection (Day 0) and at Days 3, 5, 7, and 11 of culture (Fig 6A). A mild growth (0.25 to 0.75 mm) was detected on 50% of the feathers from the neck and 100% of the feathers from the thigh (Fig 5B). The absence of growth for two feather follicles from the neck could be due to an already cornified rachis, barbs and barbules as observed by histology for a FF at day 3 of culture (Fig 3B, D3). Note that no growth was detected from Day 7, except for one feather (Fig 6A), suggesting that growth stopped mostly between 5 and 7 days of culture. Therefore, feather growth persisted upon *in vitro* cultivation in most feathers and was limited to under 1 mm and to 5–7 days.

### Expression and localization of FF marker proteins over time

**Fig 7:**
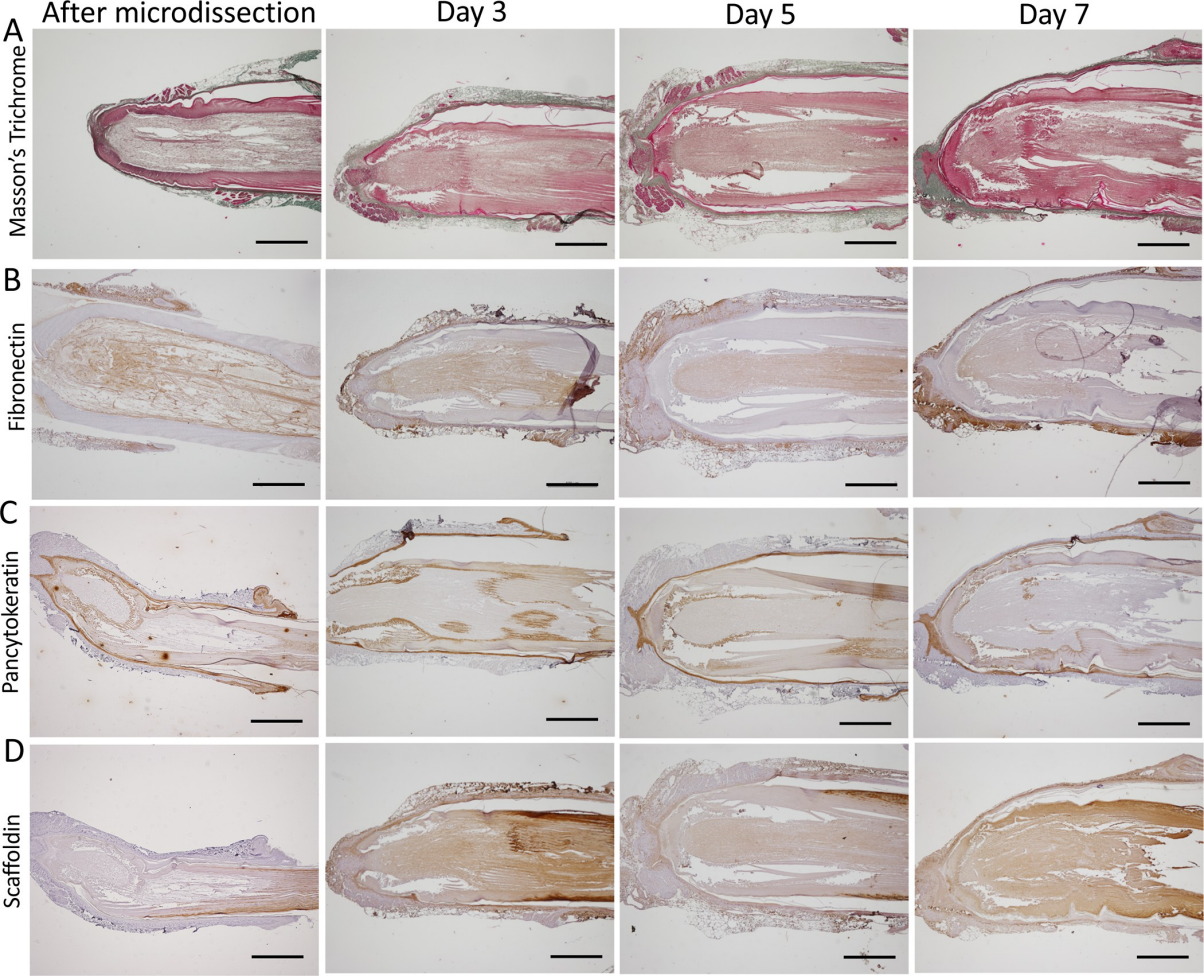
Expression and localization of avian markers by immunochemistry in FFs associated with growing feathers during cultivation *in vitro*. Formalin-fixed FFs embedded in paraffin were sectioned and stained with trichrome Masson (A), or labelled with the following antibodies: anti-fibronectin (B), anti-pan-cytokeratin (C), and anti-scaffoldin (D). Staining was performed after microdissection and at 3, 5 and 7 days of cultivation *ex vivo*. Bar represents 500 μm.

We first examined the FF after Masson’s trichrome staining, which stains collagen in bluish-green, cytoplasm in pink, and nuclei in dark red/purple. As expected, the perifollicular conjunctive tissue appeared green at all time points (Fig 7A). We next looked at the localization and expression of FF markers over culture. Fibronectin was observed in the dermis as well as in the feather pulp, and this at all time points (Fig 7B). The signal associated with fibronectin labelling was higher in dermis than in feather pulp. A slight signal was also detected in the dermal papilla, when this region was visible (*e*.*g*., Day 3). Conversely, a cytokeratin labelling was observed in all epithelial tissues. The signal was strong in the follicular wall and the epidermal collar (Fig 7C) but was weaker in the collar bulge and the feather epithelia. No signal was observed in the dermis, the dermal papilla, and the feather pulp, as expected. Lastly, we examined the expression of scaffoldin, a trichohyalin-like protein recently discovered in the scaffolding zones of growing hard skin appendages in chicken [23]. Scaffoldin is reportedly expressed in the feather sheath around growing feathers in chick embryos [23]. Here, in juvenile feathers at Day 0, we observed that scaffoldin was expressed in the feather sheath and also in barb ridges. After culture, scaffoldin labelling remained strong in feather sheath and barb ridges, but a signal of intermediate intensity was also observed in other structures (pulp, dermis, follicular wall). This was particularly visible at Day 3 and 7. We suspected that such a mild signal was non-specific or possibly linked to FF *in vitro* cultivation. Thus, we demonstrated that collagen, fibronectin, cytokeratin, and scaffoldin proteins were expressed predominantly at their proper location during culture: collagen and fibronectin were expressed in the dermis and/or feather pulp, cytokeratins in the epithelia of the follicular wall and feather, and scaffoldin in the feather sheath.

### Expression of feather follicle RNA transcripts over time in culture

**Fig 8:**
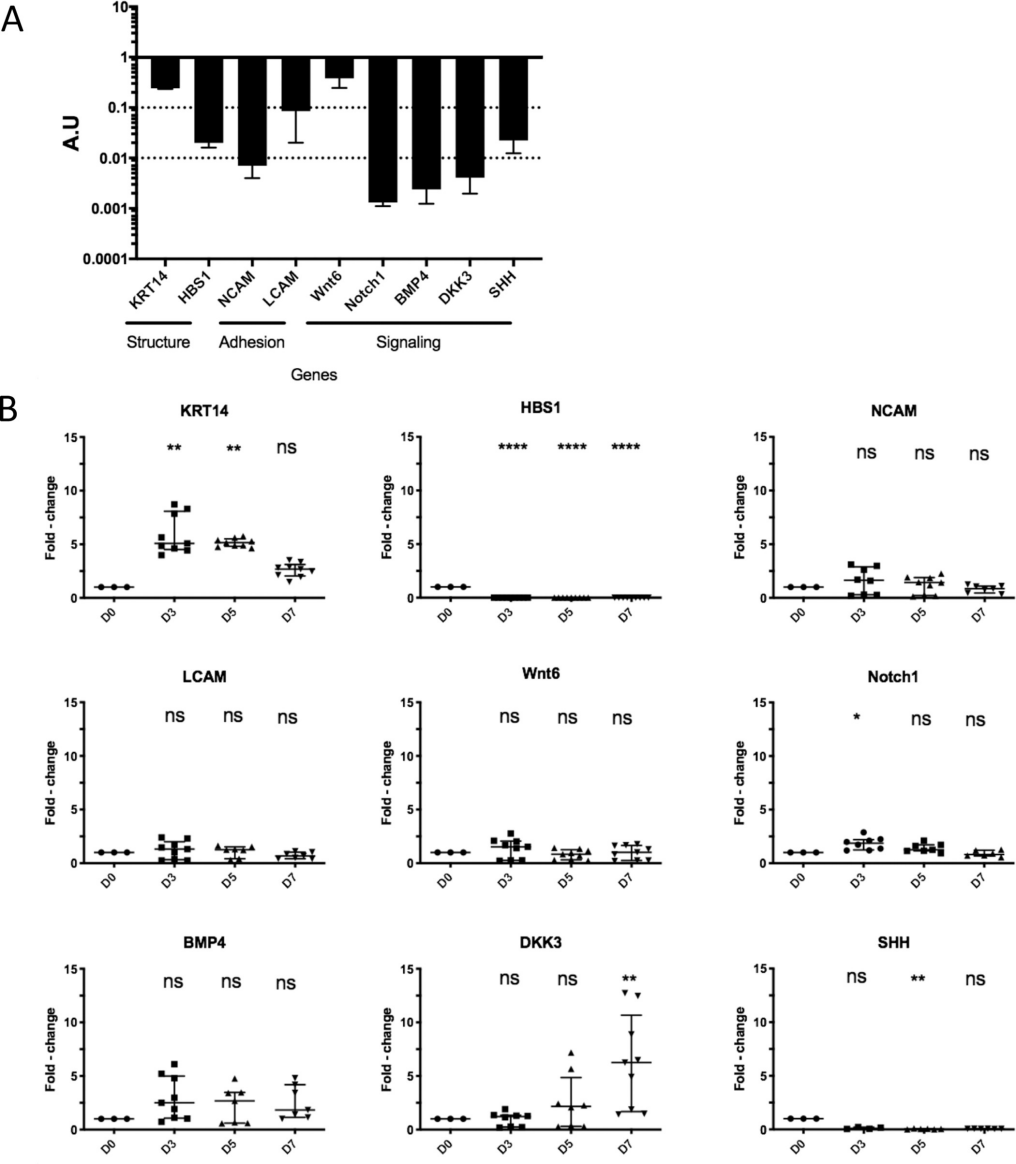
Expression of avian cellular transcripts in FFs associated to their growing feathers at microdissection and after *in vitro* cultivation. RNAs were extracted from FFs with a shortened feather after microdissection (D0), and after 3, 5, and 7 days of culture. Following cDNA synthesis, real-time qPCR was performed. At each time point, data from three samples (of 2 FF each), from 2 independent experiments, with qPCR technical repeats are shown. A. RNA levels of each gene at Day 0 were calculated relatively to the housekeeping gene RPS17 from freshly microdissected FF expressed in arbitrary units (A.U) and presented in the bar graph with median and interquartile range. B. RNA expression of the various markers was normalized to the housekeeping gene RPS17, and the fold-changes at Day 3, 5, and 7 relative to Day 0 are shown in a dot plot with median and interquartile range. No significant difference in the expression of NCAM, LCAM, Wnt6 and BMP4 was observed at any time point. In contrast, the expression of KRT14, HBS-1, Notch1, DKK3 and Shh changed significantly at several time points during the culture. Significance of differences was performed relatively to Day 0 by using the Kruskal-Wallis test with a Dunn correction for multiple comparison (adjusted p-value >0.5, ns; p-value <0.5, *; p-value<0.01, **; p-value <0.0001, ****).

Due to a limited number of antibodies recognizing cellular markers in the chicken FF and feather, we also examined RNA expression of nine genes of interest by RT-qPCR. These genes were selected because they are known to be expressed in various zones of the FF during feather regeneration of hatched chicks (see Fig 1): KRT14 (Keratin 14) is expressed in the basal layer of the follicular wall and feather epithelium [32]; NCAM (neural cell adhesion molecule) is expressed mostly in the dermal papilla [17]; LCAM (liver cell adhesion molecule) in the feather epithelium [17]; Wnt6 (wingless integrated gene 6) in the epithelial ramogenic zone and follicular wall (at lower levels) [17]; Notch1 in dermal papilla, feather pulp, and epithelial ramogenic zone [17]; BMP4 (bone morphogenetic protein 4) in dermal papilla, feather pulp, and barb ridges [4, 17]; DKK3 (Dickkopf 3) mostly in the dermal papilla and feather pulp [17]; Shh (sonic hedgehog) in the barb ridges (in the marginal plates) of the growing feather [4, 33]; and HBS1 keratin (HBS, hard basic sauropsid-specific) in the barb ridges [14]. We first examined the RNA expression levels of each gene (relative to the expression of the reference gene RPS17), on freshly microdissected FF. Note that each of the genes showed a lower expression than RPS17 (Fig 8A). Genes were classified in three categories according to their expression level: high, between 1 and 10^−1^ A.U. (KRT14 and Wnt6); medium, between 10^−1^ and 10^−2^ A.U. (HBS1, LCAM, Shh), and low, <10^−2^ A.U. (NCAM, Notch1, BMP4, and DKK3).

To examine the molecular modifications in the FF over cultivation *in vitro*, we next examined the RNA expression of our selected markers at Day 3, 5, and 7 of cultivation relative to Day 0. The average quantifiable yield of RNA per 2 FF (on 3 independent samples) was of 417 ng at Day 0, 46 ng at Day 3, 71 ng at Day 5, and 64 ng at Day 7, indicating a reduction in transcription activity during cultivation. Sufficient material was nevertheless available for transcript analysis. The transcript expression of NCAM, LCAM, Wtn6 and BMP4 remained virtually unchanged upon culture until Day 7 (Fig 8B). In contrast, Shh and HBS1 expression substantially decreased from Day 3, DKK3 expression increased 6.28-fold at Day 7, Notch1 expression increased slightly at Day 3, and KRT14 expression transiently increased at Day 3 and 5 by about 5-fold (Fig 8B). These data show that the cultivation globally reduced RNA transcription activity in the FF and modified the expression levels of four of the nine genes examined, while the others were maintained. The significance of these results will be discussed below.

## Discussion

In this study, we showed by microscopic analyses that the integrity of the FF was fully preserved morphologically for as long as 7 days of cultivation, except in the feather pulp. In the feather pulp, signs of cell degeneration were visible after 1 day of culture. *In vivo*, the feather pulp is supplied in nutrients and oxygen by a prominent single axial artery. The degeneration of feather pulp cells in culture may be the consequence of a brutal deprivation of blood supply [34]. Because the growing feather is covered by the feather shaft which is non-permeable, nutrients entry by diffusion may be not be adequate to feed pulp cells at the feather’s center. Moreover, we did not observe the replenishing of pulp cells via the dermal papilla. This may be due to high levels of Wnt6 transcripts over time in culture, which have been shown to suppressed pulp formation [17].

The preservation of the feather epithelium can be explained by the fact that this pluri-stratified epithelium is an avascular region, which is physiologically fed by diffusion. Therefore, nutrients reduction might be less deleterious to some extents than for the pulp. The follicular wall cells may also have benefited from simple diffusion through the culture medium for their survival. In addition, the upper layers of the FF epithelium (*e*.*g*., feather sheath) are highly keratinized zones made of corneocytes, which are dead cells and therefore not dependent of nutrients. The results of the TEM analyses, mostly performed at the follicular wall level, confirmed the histologic observations showing that the epithelia are intact. Indeed, the epithelium ultrastructure showed no or minor changes in intracellular organelles (mitochondria) or cellular structures (desmosomes) morphology until Day 7, with rare necrotic cells. Importantly, the integrity of desmosomes was consistent with the epithelial cell cohesion.

In this study, the expression of twelve markers was confirmed in FF harboring juvenile growing feather freshly microdissected from 3 week-old WL chickens. Collagen was localized in the dermis surrounding the follicular wall, as demonstrated by Masson’s trichrome staining. Collagen from the dermal papilla (col6a1 and 18a1) and feather pulp (col4a1) cannot be visualized with this staining, as already reported [17]. Three other structural proteins (fibronectin, keratin, and scaffoldin) were found at their expected locations by immunochemistry, and were stable over time. Here, scaffoldin has been observed in the feather sheath as reported earlier for 18-day old embryos [23], but also within the barb ridges. Such a localization although not depicted yet is compatible with Alibardi’s previous data showing the presence of "periderm granules" in supportive cells ("barb vane ridge cells" or "wedge cells") located between growing barbules [35]. In another study, Alibardi showed by immunogold labeling that periderm granules contain scaffoldin [36]. The presumed role of scaffoldin being is form a transient scaffold in growing feathers [36, 37]. Thus, additional experiments will be needed to determine whether scaffoldin is located in barb vane ridge cells of juvenile feathers as we can expect. Among the nine genes quantified by RT-qPCR, Wnt6 transcript was the most expressed after FF microdissection. Wnt6 has been shown to be highly expressed in the feather epithelium of regenerating feathers by *in situ* hybridization [17]. The high expression of Wnt6 is in accordance with the growing activity of the FF at that time [17, 38]. It is noticeable that the other signaling molecules (BMP4, Notch1, DKK3, and Shh) were moderately to poorly expressed in comparison to Wnt6. KRT14 was another highly expressed gene after microdissection. This was not surprising as this gene encodes a major cytoskeleton protein from the basal layer of the follicular wall and the adult feather epithelium [32, 39]. LCAM was expressed about ten-fold more than NCAM. LCAM is known to be expressed in the feather epithelium, while NCAM is expressed mostly in the dermal papilla/dermal sheath and weakly the feather branching epithelium [17]. It is therefore not surprising that LCAM was more expressed than NCAM, considering the relative surface areas marked into the FF for each gene.

Interestingly, expression of eight of these markers in the FF was maintained over the cultivation time, both in term of their protein localization (fibronectin, alphakeratins, and scaffoldin) and their mRNA expression levels (NCAM, LCAM, Wnt6, Notch1, and BMP4) relatively to the housekeeping gene (RPS17). This indicates that part of the biological processes and structure at the molecular level is well preserved in the cultured FF. However, we observed some modifications in the expression levels of four marker genes encoding two signaling molecules (Shh, DKK3) and two keratins (KRT14, HBS1). Shh expression was drastically reduced from 3 days of culture. Shh is essential for inducing apoptosis of the marginal plates and differentiation of the barbule plates of the feather epithelia [4, 33, 40]. Moreover, downregulation of Shh transcript is associated with alterations of feather branching in regenerating feathers after treatment of 6-month old chickens with chemotherapeutic agents [41]. Therefore, we speculated that the differentiation program leading to barbs and barbules formation was abrogated or altered from Day 3 of culture. Conversely, DKK3 expression increased progressively from Day 5. In regenerating feathers, DKK3 is expressed at moderate levels in the dermal papilla (by *in situ* hybridization) as well as in the feather pulp (by RT-qPCR) [17]. Unlike DKK2, DKK3 is not a Wnt inhibitor and its overexpression does not disrupt feather regeneration or lead to an obvious phenotype [17]. Therefore, the reason why the DKK3 gene was overexpressed in our study and its potential effect remain unclear. KRT14 is mainly expressed in basal keratinocytes of the follicular wall and feather epithelia [32, 39]. Herein, we observed that KRT14 gene expression was transiently upregulated at Day 3 and 5 of culture. We therefore hypothesize that proliferation of basal keratinocytes may have occurred for several days during cultivation, or that the keratinocytes differentiation was impaired. Lastly, HBS1 transcript expression became undetectable from 3 days of culture. HBS1 keratin is a hard basic (type II) sauropsid-specific keratin, which was localized by immunostaining in the cornifying epithelial cells of feathers [14]. The HBS1 transcription shut-down that we observed upon cultivation suggests an impairment of the cornification process in feathers. Although the structure of the FF was preserved based on certain markers of expression and on morphological criteria, the drastic reduction in Shh and HBS1 transcription and the upregulation of KRT14 suggest an impairment of the feather cornification process and/or barb ridge differentiation.

We observed a slight growth of the feathers after FF cultivation *in vitro*, which was limited to 1 mm over 7 days. The slow growth of feathers in culture may be due to the fact that microdissected FFs were in their late stage of growth, or due to the modifications in gene expression. Indeed, part of our results are compatible with an impairment of the cornification process and/or barb ridge differentiation (as discussed above), two phenomena that may have contributed to the limited feather growth. Lastly, the growth of feather or feather renewal is known to be critically dependent onto nutrients such as sulphur amino-acids [42], since feather proteins have a high cysteine content [43]. Although we renewed the culture medium every 2 days, it is possible that the limited growth observed was due to a lack in certain amino-acids. A lack in vitamins (*e*.*g*., acid ascorbic) may also be involved in this slow growth. Indeed, in human HF, addition of a L-ascorbic acid derivative induces the secretion of the insulin-like growth factor 1 in dermal papilla cells and subsequently the proliferation of follicular keratinocytes with a better hair shaft growth [44]. Although the role of insulin and acid ascorbic in feather growth *in vivo* remains very poorly understood [45], the benefit of ascorbic acid supplementation in FF culture could be interesting to investigate.

The structure and morphogenesis mechanisms, in term of spatio-temporal cell differentiation, are more complex for feathers [2, 5, 7, 46, 47] than for hairs [16]. For example, the FFs of a chicken generate feathers of different stiffness with variable cornified structures, depending among other things on the types of CBP, proteins which are prevalent proteins in adult feathers [39, 46, 48]. In addition, pennaceous feathers predominant in adult chickens, display a regular epithelium and a two-level branched cornified epithelium (barbs and barbules) attached to a central axis (the rachis). Such organization is based on barb ridge formation and tightly regulated differentiation processes, in which specific cells undergo either apoptosis or cornification [40, 46, 49]. In contrast, the hair shaft originates from an unbranched epithelium, which only get keratinized over differentiation [50]. For these reasons, it is therefore not surprising that maintaining pathways of growth and differentiation *in vitro* could be more complex for feathers than for hairs.

This model is the first one of FF cultivation *in vitro*. Compare to previous chicken skin models such as embryonic skin explants [25–27] or skin equivalents [51], the model herein is the only one that exhibits invaginated FF from juvenile feathers wherease embryonic skin explants that show feather buds only. As the development of *in vitro* HF models in the past decade (reviewed in [52]) has reduced experimentation on rabbits and mice, the new *in vitro* FF culture model we have developed herein could reduce experimentation on chickens. For the moment, this technique cannot replace the use of chickens as we need chickens as donors. The FF culture system may prove useful to study the acute susceptibility of feathers to toxins (*e*.*g*., mycotoxins) [53, 54], and to study host-pathogens interactions for avian viruses that replicate in the FF, such as Marek’s disease virus [55], avian leukosis virus [56], chicken anemia virus [57], and avian influenza viruses [58]. This model could help characterize mutant viruses, study viral morphogenesis and cellular responses, and test anti-viral strategies in a physiologically relevant complex cellular system. Note that for the moment, this system is limited to applications feasible in a period of seven days and not requiring high cell proliferation.

In conclusion, we demonstrate herein that the model initially developed by Westgate for HF [20] is effective to cultivate structurally intact chicken FF for 7 days.

## Supporting information

S1 Fig Histological structure of feather follicles from microdissection to day 13 of cultivation *in vitro*. Formalin-fixed FF embedded in paraffin were sectioned and stained with HES. Structure after microdissection (D0), day 3 (D3), day 5 (D5), day 7 (D7), day 11 (D11) and day 13 (D13) of culture *in vitro*. From day 3 of cultivation, we observed necrosis of the feather pulp (*) and of the inner sheath, with pyknotic nuclei from necrotic cells scattered mainly in the dermis (black arrow). Number of necrotic cells progressively increased with time of cultivation and at day 11, most follicular cells appeared necrotic. Note the preservation of the dermal papillae (dp). At day 13, some mineral cristae were visible (white arrowhead).
(TIF)

S1 File Minimal data set of quantitative data from Figs 6 and 8. (PDF)

## Abbreviations

BMP: bone morphogenetic protein
DKK3: Dickkopf 3
ERZ: epithelial ramogenic zone
FF: feather follicle
HBS: hard basic sauropsid-specific
HES: hematoxylin-eosin-safranin
HF: hair follicle
KRT14: keratin 14
qPCR: quantitative PCR
RT-qPCR: reverse-transcription qPCR
Shh: sonic hedgehog
TEM: transmission electron microscopy
Wnt6: wingless integrated gene 6
