# Supplementary figures and images for: Establishment of a culture model for the prolonged maintenance of chicken feather follicles structure in vitro
Source: PLoS One. 2022 Oct 7;17(10):e0271448. doi: 10.1371/journal.pone.0271448 (PMC9544018; doi:10.1371/journal.pone.0271448)

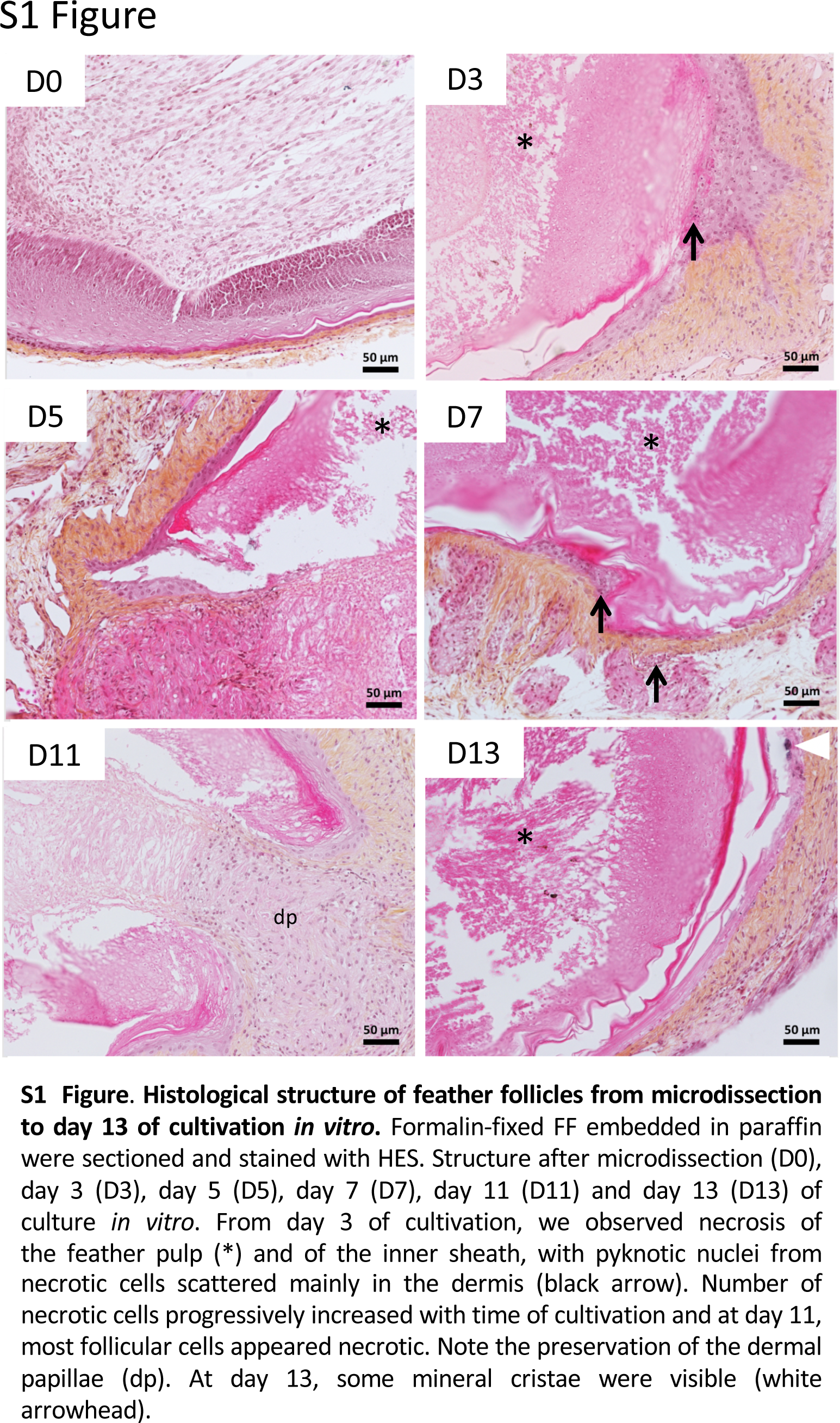

Supplement: S1 Fig — Formalin-fixed FF embedded in paraffin were sectioned and stained with HES. Structure after microdissection (D0), day 3 (D3), day 5 (D5), day 7 (D7), day 11 (D11) and day 13 (D13) of culture in vitro. From day 3 of cultivation, we observed necrosis of the feather pulp (*) and of the inner sheath, with pyknotic nuclei from necrotic cells scattered mainly in the dermis (black arrow). Number of necrotic cells progressively increased with time of cultivation and at day 11, most follicular cells appeared necrotic. Note the preservation of the dermal papillae (dp). At day 13, some mineral cristae were visible (white arrowhead). (TIF) [file pone.0271448.s001.tif]
