# Supplementary material for: Establishment of a culture model for the prolonged maintenance of chicken feather follicles structure in vitro
Source: PLoS One. 2022 Oct 7;17(10):e0271448. doi: 10.1371/journal.pone.0271448 (PMC9544018; doi:10.1371/journal.pone.0271448)
Supplement: S1 File — (PDF) [file pone.0271448.s002.pdf]

Data Figure 6

	Size in mm				
**Follicles**	**D0**	**D3**	**D5**	**D7**	**D11**
Neck#1	11,5	11,5	11,5	11,5	11,5
Neck#2	6	6,25	6,5	6,5	6,5
Neck#3	10,5	10,5	10,5	10,5	10,5
Neck#4	10,25	10,5	10,75	11	11
Thigh#1	10,5	10,75	10,75	10,75	10,75
Thigh#2	6,25	6,5	6,5	6,5	6,5
Thigh#3	10	10	10	10	10,25
Thigh#4	9	9	9,25	9,5	9,5

Data Figure 8A

Marker	Day	Trial	Chicken	Sample	RNA levels (A.U)
**KRT14**	D0	T1	C1	S1	0,3276
	D0	T1	C1	S1	0,3186
	D0	T1	C1	S1	0,2717
	D0	T2	C2	S2	0,2636
	D0	T2	C2	S2	0,2112
	D0	T2	C2	S2	0,2547
	D0	T2	C2	S3	0,2349
	D0	T2	C2	S3	0,2333
	D0	T2	C2	S3	0,2316
**HBS1**	D0	T1	C1	S1	0,0318
	D0	T1	C1	S1	0,0391
	D0	T2	C2	S2	0,0146
	D0	T2	C2	S2	0,0150
	D0	T2	C2	S2	0,0190
	D0	T2	C2	S3	0,0201
	D0	T2	C2	S3	0,0175
	D0	T2	C2	S3	0,0226
**NCAM**	D0	T1	C1	S1	0,0049
	D0	T1	C1	S1	0,0035
	D0	T1	C1	S1	0,0038
	D0	T2	C2	S2	0,0064
	D0	T2	C2	S2	0,0074
	D0	T2	C2	S2	0,0074
	D0	T2	C2	S3	0,0080
	D0	T2	C2	S3	0,0075
	D0	T2	C2	S3	0,0097
**LCAM**	D0	T1	C1	S1	0,0194
	D0	T1	C1	S1	0,0207
	D0	T2	C2	S2	0,0793
	D0	T2	C2	S2	0,0976
	D0	T2	C2	S3	0,0747
	D0	T2	C2	S3	0,0913
	D0	T2	C2	S3	0,0913
**Wnt6**	D0	T1	C1	S1	0,5322
	D0	T1	C1	S1	0,7220
	D0	T1	C1	S1	0,5249
	D0	T2	C2	S2	0,3577
	D0	T2	C2	S2	0,3291
	D0	T2	C2	S2	0,4622
	D0	T2	C2	S3	0,2365
	D0	T2	C2	S3	0,2365
	D0	T2	C2	S3	0,2606
**Notch1**	D0	T1	C1	S1	0,0012
	D0	T1	C1	S1	0,0010
	D0	T2	C2	S2	0,0014
	D0	T2	C2	S2	0,0013
	D0	T2	C2	S3	0,0015
	D0	T2	C2	S3	0,0015
**BMP4**	D0	T1	C1	S1	0,0038
	D0	T1	C1	S1	0,0039
	D0	T1	C1	S1	0,0035

	D0	T2	C2	S2	0,0009
	D0	T2	C2	S2	0,0011
	D0	T2	C2	S2	0,0011
	D0	T2	C2	S3	0,0013
	D0	T2	C2	S3	0,0012
**DKK3**	D0	T1	C1	S1	0,0104
	D0	T1	C1	S1	0,0084
	D0	T2	C2	S2	0,0039
	D0	T2	C2	S2	0,0041
	D0	T2	C2	S2	0,0042
	D0	T2	C2	S3	0,0022
	D0	T2	C2	S3	0,0021
	D0	T2	C2	S3	0,0016
**SHH**	D0	T1	C1	S1	0,0245
	D0	T1	C1	S1	0,0235
	D0	T1	C1	S1	0,0191
	D0	T2	C2	S2	0,0299
	D0	T2	C2	S2	0,0332
	D0	T2	C2	S2	0,0291
	D0	T2	C2	S3	0,0144
	D0	T2	C2	S3	0,0107

Data Figure 8B

Marker	Day	Trial	Chicken	Set	F_Change
**NCAM**	D0-D3	T1	C1	S1	0,310
	D0-D3	T1	C1	S1	0,283
	D0-D3	T1	C1	S1	0,203
	D0-D3	T2	C2	S2	3,109
	D0-D3	T2	C2	S2	2,615
	D0-D3	T2	C2	S2	2,983
	D0-D3	T2	C2	S3	1,693
	D0-D3	T2	C2	S3	1,591
	D0-D5	T1	C1	S1	0,176
	D0-D5	T1	C1	S1	0,137
	D0-D5	T1	C1	S1	0,265
	D0-D5	T2	C2	S2	1,914
	D0-D5	T2	C2	S2	1,862
	D0-D5	T2	C2	S2	2,261
	D0-D5	T2	C2	S3	1,467
	D0-D5	T2	C2	S3	1,437
	D0-D5	T2	C2	S3	1,184
	D0-D7	T1	C1	S1	0,334
	D0-D7	T1	C1	S1	0,453
	D0-D7	T2	C2	S2	0,857
	D0-D7	T2	C2	S2	0,772
	D0-D7	T2	C2	S2	1,107
	D0-D7	T2	C2	S3	1,245
	D0-D7	T2	C2	S3	1,062
**LCAM**	D0-D3	T1	C1	S1	0,260
	D0-D3	T1	C1	S1	0,394
	D0-D3	T1	C1	S1	0,258
	D0-D3	T2	C2	S2	1,713
	D0-D3	T2	C2	S2	2,406
	D0-D3	T2	C2	S2	2,308
	D0-D3	T2	C2	S3	1,030
	D0-D3	T2	C2	S3	1,323
	D0-D3	T2	C2	S3	1,467
	D0-D5	T1	C1	S1	0,303
	D0-D5	T1	C1	S1	0,423
	D0-D5	T2	C2	S2	1,237
	D0-D5	T2	C2	S2	1,533
	D0-D5	T2	C2	S2	1,632
	D0-D5	T2	C2	S3	1,254
	D0-D5	T2	C2	S3	1,307
	D0-D7	T1	C1	S1	0,407
	D0-D7	T1	C1	S1	0,504
	D0-D7	T1	C1	S1	0,424
	D0-D7	T2	C2	S2	0,686
	D0-D7	T2	C2	S2	0,857
	D0-D7	T2	C2	S3	1,117
	D0-D7	T2	C2	S3	1,035
**KRT14**	D0-D3	T1	C1	S1	5,086
	D0-D3	T1	C1	S1	4,428
	D0-D3	T1	C1	S1	4,616
	D0-D3	T2	C2	S2	3,991
	D0-D3	T2	C2	S2	4,846
	D0-D3	T2	C2	S2	5,644
	D0-D3	T2	C2	S3	7,853
	D0-D3	T2	C2	S3	8,301
	D0-D3	T2	C2	S3	8,714
	D0-D5	T1	C1	S1	5,157
	D0-D5	T1	C1	S1	4,879
	D0-D5	T1	C1	S1	5,762
	D0-D5	T2	C2	S2	5,278
	D0-D5	T2	C2	S2	5,389
	D0-D5	T2	C2	S2	5,618
	D0-D5	T2	C2	S3	4,757
	D0-D5	T2	C2	S3	4,891
	D0-D5	T2	C2	S3	4,627
	D0-D7	T1	C1	S1	2,875
	D0-D7	T1	C1	S1	2,485
	D0-D7	T1	C1	S1	2,682
	D0-D7	T2	C2	S2	1,498
	D0-D7	T2	C2	S2	1,950
	D0-D7	T2	C2	S2	2,134
	D0-D7	T2	C2	S3	2,822
	D0-D7	T2	C2	S3	3,523
	D0-D7	T2	C2	S3	3,356
**Wnt6**	D0-D3	T1	C1	S1	0,267
	D0-D3	T1	C1	S1	0,232
	D0-D3	T1	C1	S1	0,267
	D0-D3	T2	C2	S2	2,028

	D0-D3	T2	C2	S2	2,770
	D0-D3	T2	C2	S2	2,099
	D0-D3	T2	C2	S3	1,411
	D0-D3	T2	C2	S3	1,533
	D0-D3	T2	C2	S3	1,678
	D0-D5	T1	C1	S1	0,288
	D0-D5	T1	C1	S1	0,231
	D0-D5	T1	C1	S1	0,354
	D0-D5	T2	C2	S2	1,157
	D0-D5	T2	C2	S2	1,347
	D0-D5	T2	C2	S2	1,434
	D0-D5	T2	C2	S3	0,849
	D0-D5	T2	C2	S3	0,689
	D0-D5	T2	C2	S3	0,961
	D0-D7	T1	C1	S1	0,252
	D0-D7	T1	C1	S1	0,210
	D0-D7	T1	C1	S1	0,250
	D0-D7	T2	C2	S2	1,016
	D0-D7	T2	C2	S2	1,304
	D0-D7	T2	C2	S2	0,831
	D0-D7	T2	C2	S3	1,778
	D0-D7	T2	C2	S3	1,682
	D0-D7	T2	C2	S3	1,636
**DKK3**	D0-D3	T1	C1	S1	0,233
	D0-D3	T1	C1	S1	0,279
	D0-D3	T1	C1	S1	0,197
	D0-D3	T2	C2	S2	1,275
	D0-D3	T2	C2	S2	1,905
	D0-D3	T2	C2	S2	1,357
	D0-D3	T2	C2	S3	1,154
	D0-D3	T2	C2	S3	1,298
	D0-D5	T1	C1	S1	0,371
	D0-D5	T1	C1	S1	0,277
	D0-D5	T1	C1	S1	0,291
	D0-D5	T2	C2	S2	2,417
	D0-D5	T2	C2	S2	2,075
	D0-D5	T2	C2	S2	2,271
	D0-D5	T2	C2	S3	5,670
	D0-D5	T2	C2	S3	7,177
	D0-D7	T1	C1	S1	1,886
	D0-D7	T1	C1	S1	1,419
	D0-D7	T1	C1	S1	1,479
	D0-D7	T2	C2	S2	6,483
	D0-D7	T2	C2	S2	6,262
	D0-D7	T2	C2	S2	4,913
	D0-D7	T2	C2	S3	12,467
	D0-D7	T2	C2	S3	12,729
	D0-D7	T2	C2	S3	8,877
**SHH**	D0-D3	T2	C2	S2	0,217
	D0-D3	T2	C2	S2	0,169
	D0-D3	T2	C2	S3	0,054
	D0-D3	T2	C2	S3	0,038
	D0-D5	T1	C1	S1	0,010
	D0-D5	T1	C1	S1	0,010
	D0-D5	T2	C2	S2	0,089
	D0-D5	T2	C2	S2	0,061
	D0-D5	T2	C2	S3	0,044
	D0-D5	T2	C2	S3	0,042
	D0-D7	T2	C2	S2	0,051
	D0-D7	T2	C2	S2	0,044
	D0-D7	T2	C2	S2	0,061
	D0-D7	T2	C2	S3	0,065
	D0-D7	T2	C2	S3	0,072
	D0-D7	T2	C2	S3	0,060
**BMP4**	D0-D3	T1	C1	S1	0,735
	D0-D3	T1	C1	S1	1,026
	D0-D3	T1	C1	S1	1,122
	D0-D3	T2	C2	S2	6,105
	D0-D3	T2	C2	S2	5,205
	D0-D3	T2	C2	S2	4,790
	D0-D3	T2	C2	S3	2,500
	D0-D3	T2	C2	S3	2,979
	D0-D3	T2	C2	S3	1,921
	D0-D5	T1	C1	S1	0,610
	D0-D5	T1	C1	S1	0,581
	D0-D5	T1	C1	S1	0,663
	D0-D5	T2	C2	S2	3,466
	D0-D5	T2	C2	S2	4,768
	D0-D5	T2	C2	S3	2,679
	D0-D5	T2	C2	S3	3,414
	D0-D7	T1	C1	S1	1,002

	D0-D7	T1	C1	S1	1,143
	D0-D7	T2	C2	S2	1,421
	D0-D7	T2	C2	S2	1,836
	D0-D7	T2	C2	S3	3,426
	D0-D7	T2	C2	S3	4,812
	D0-D7	T2	C2	S3	4,189
**Notch1**	D0-D3	T1	C1	S1	1,188
	D0-D3	T1	C1	S1	1,204
	D0-D3	T1	C1	S1	1,374
	D0-D3	T2	C2	S2	1,998
	D0-D3	T2	C2	S2	2,112
	D0-D3	T2	C2	S2	2,885
	D0-D3	T2	C2	S3	2,240
	D0-D3	T2	C2	S3	1,733
	D0-D5	T1	C1	S1	1,623
	D0-D5	T1	C1	S1	1,715
	D0-D5	T1	C1	S1	2,112
	D0-D5	T2	C2	S2	1,258
	D0-D5	T2	C2	S2	1,174
	D0-D5	T2	C2	S3	0,927
	D0-D5	T2	C2	S3	1,157
	D0-D7	T1	C1	S1	1,203
	D0-D7	T1	C1	S1	1,263
	D0-D7	T2	C2	S2	0,724
	D0-D7	T2	C2	S2	0,917
	D0-D7	T2	C2	S3	0,730
	D0-D7	T2	C2	S3	0,554
**HBS1**	D0-D3	T1	C1	S1	NA
	D0-D3	T1	C1	S1	NA
	D0-D3	T1	C1	S1	NA
	D0-D3	T2	C2	S2	NA
	D0-D3	T2	C2	S2	NA
	D0-D3	T2	C2	S2	NA
	D0-D3	T2	C2	S3	NA
	D0-D3	T2	C2	S3	NA
	D0-D3	T2	C2	S3	NA
	D0-D5	T1	C1	S1	NA
	D0-D5	T1	C1	S1	NA
	D0-D5	T1	C1	S1	NA
	D0-D5	T2	C2	S2	NA
	D0-D5	T2	C2	S2	NA
	D0-D5	T2	C2	S2	NA
	D0-D5	T2	C2	S3	NA
	D0-D5	T2	C2	S3	NA
	D0-D5	T2	C2	S3	NA
	D0-D7	T1	C1	S1	NA
	D0-D7	T1	C1	S1	NA
	D0-D7	T1	C1	S1	NA
	D0-D7	T2	C2	S2	NA
	D0-D7	T2	C2	S2	NA
	D0-D7	T2	C2	S2	NA
	D0-D7	T2	C2	S3	NA
	D0-D7	T2	C2	S3	NA
	D0-D7	T2	C2	S3	NA
